# Overcoming barriers to early diagnosis and treatment of p.Val142Ile amyloid transthyretin (ATTR) cardiomyopathy

**DOI:** 10.1007/s10741-026-10651-1

**Published:** 2026-07-02

**Authors:** Keith C. Ferdinand, Senthil Selvaraj, Kevin M. Alexander, Saurabh Malhotra

**Affiliations:** 1 Division of Cardiology, Tulane University School of Medicine, 1430 Tulane Avenue, New Orleans, LA 70112 USA; 2 Duke Molecular Physiology Institute and the Division of Cardiology, Duke University School of Medicine, Durham, NC USA; 3 Stanford Amyloid Center, Stanford University School of Medicine, Palo Alto, CA USA; 4 Division of Cardiology, Cook County Health, Chicago, IL USA; 5 Division of Cardiology, Rush Medical College, Chicago, IL USA

**Keywords:** ATTR amyloidosis, Cardiomyopathy, Diagnosis, Treatment, p.Val142Ile variant

## Abstract

The most common hereditary transthyretin (ATTRv) amyloidosis variant in the United States, *Val122Ile* (p.Val142Ile), is predominantly detected in populations of African ancestry. In this narrative review, we highlight the current challenges and discuss priority focus areas to improve the timely diagnosis and treatment of p.Val142Ile ATTRv amyloidosis across populations. Studies suggest geographic, economic, and socio-economic discordance in timely diagnosis, treatment access, and outcomes in regions with a high proportion of at‑risk individuals. Many ATTR amyloidosis symptoms overlap with other diseases due to their nonspecific and heterogenous nature. Consequently, optimized screening tools for biomarker identification and specialty care services/networks are required to determine at-risk individuals and improve clinical outcomes. However, access to genetic screening can be limited and diagnosis is complicated by variable p.Val142Ile ATTRv amyloidosis penetrance coupled with phenotypic evolution. To enhance existing treatment guidelines, regular monitoring of “at-risk” patients, multidisciplinary management, and purposeful clinical trial recruitment of minority populations are recommended.

**Graphical Abstract:**

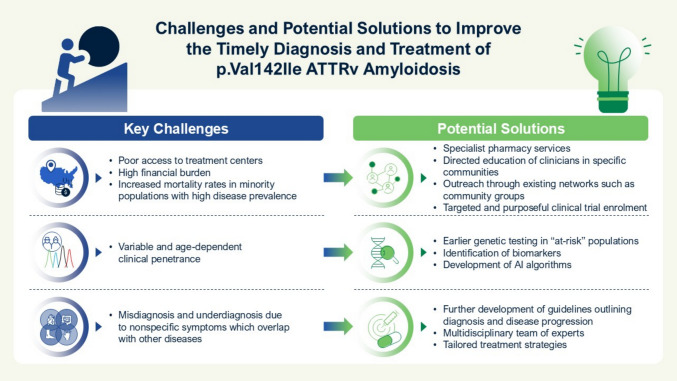

## Introduction

Transthyretin (ATTR) amyloidosis is a disorder characterized by the progressive deposition of insoluble misfolded transthyretin (TTR) protein that forms amyloid fibrils in multiple organs and tissues [1–4]. ATTR amyloidosis presents most frequently as cardiomyopathy (ATTR-CM) or polyneuropathy (ATTR-PN). ATTR-CM can lead to heart failure, arrhythmias, and death, while ATTR-PN may manifest as autonomic dysfunction with dissociated sensory disturbance in early-onset small fiber neuropathy (early onset), or as sensorimotor symptoms due to larger fiber involvement in late-onset disease. However, ATTR amyloidosis may also present as a mixed clinical phenotype (ATTR mixed) [4–8].

TTR deposition can be caused by destabilization of the wild-type TTR protein known as wild-type transthyretin (ATTRwt) amyloidosis or may be hereditary transthyretin (ATTRv) amyloidosis [4]. The Val122Ile (p.Val142Ile) substitution is the most common ATTR variant detected in the United States (US), with an estimated 1.5 million carriers [9–11]. Of these carriers, nearly one third are over the age of 50 years and are therefore at risk of disease penetrance [12], with the typical age of onset for carriers of the p.Val142Ile variant estimated to be ≥ 55 years [13, 14]. Individuals harboring this variant are predominantly of African ancestry, with carrier rates of 3–11% in this population [15–17]. Given its predominant association with cardiomyopathy, cardiac investigations – including electrocardiography, echocardiography, cardiac biomarkers, cardiac magnetic resonance imaging, and scintigraphy – are recommended to be considered [13]. However, patients may also present with neurologic, renal, or mixed manifestations, which should be recognized when considering the full clinical spectrum of the disease [9, 18, 19].

ATTRv-CM p.Val142Ile carrier status is associated with an increased prevalence of atrial fibrillation/flutter, heart failure, and carpal tunnel syndrome compared with individuals not carrying this variant [16, 20–25]. A large study conducted in the United Kingdom (UK) in ~ 3,000 patients with suspected ATTR-CM has shown that a substantial increase in specialist referrals and earlier diagnosis of ATTR-CM over a 20-year period was accompanied by a significant improvement in survival, even in patients with p.Val142Ile ATTRv-CM [26]. However, diagnosis of ATTR-CM (including that associated with the p.Val142Ile variant) is often delayed, and this could be attributed to the nonspecific and heterogeneous nature of symptoms [26–30]. Patients may endure multiple medical evaluations, incorrect diagnoses, and inappropriate treatment prior to the identification of ATTR-CM [26–28]. Such issues with achieving a prompt and accurate diagnosis remain a barrier to better outcomes for patients with ATTR-CM [27, 28].

For individuals carrying a *TTR* mutation, current guidelines recommend initiating annual follow-up 10 years before the predicted age of disease onset, with surveillance frequency increasing as carriers approach that age [13, 14]. The choice of investigations should be tailored to the specific mutation, reflecting the heterogenous phenotypes associated with different *TTR* variants [13, 14]. Carriers should also be educated about the symptoms most relevant to their mutation, and surveillance intervals shortened if features suggestive of ATTR amyloidosis emerge [13]. Minimum diagnostic criteria for symptomatic disease in carriers include: 1) at least one quantified or objective symptom or sign of ATTR amyloidosis; 2) a symptom likely related to ATTR amyloidosis in the absence of objective signs plus an abnormal test result; and 3) two abnormal test results in the absence of symptoms [13].

This narrative review aims to summarize the evidence, best practices, and potential solutions to improve timely diagnosis and treatment of p.Val142Ile ATTRv-CM, with a focus on equitable access across populations.

## Methods

A literature search was conducted to identify relevant studies and articles that reported on themes including the epidemiology, clinical presentation, patient diagnostic journey, and disease progression for patients with ATTR amyloidosis, with a focus on patients with p.Val142Ile ATTRv-CM.

### Search strategy and eligibility criteria

Searches of the electronic database PubMed were conducted from November to December 2024, and repeated in February 2026 to capture newly published articles since the original searches. The search terms used for each theme are provided in Table 1. The search was restricted to English-language publications, with no date limits applied.

**Table 1:** Search terms used to identify worldwide and US-specific literature on ATTR amyloidosis and the p.Val142Ile variant

Topic	Search terms
Epidemiology	
ATTR amyloidosis	
Worldwide	((Amyloidosis[Title/Abstract]) AND ((ATTR[Title/Abstract]) OR (TTR[Title/Abstract]))) AND ((((Epidemiology[Title]) OR (prevalence[Title])) OR (Incidence[Title])) OR (Penetrance[Title]))
US	(((Amyloidosis[Title/Abstract]) AND ((ATTR[Title/Abstract]) OR (TTR[Title/Abstract]))) AND ((((Epidemiology[Title]) OR (prevalence[Title])) OR (Incidence[Title])) OR (Penetrance[Title]))) AND (((American[Title/Abstract]) OR (America[Title/Abstract])) OR (United States[Title/Abstract]))
p.Val142Ile variant-specific	
Worldwide	(((Amyloidosis[Title/Abstract]) AND ((ATTR[Title/Abstract]) OR (TTR[Title/Abstract]))) AND ((((Epidemiology[Title]) OR (prevalence[Title])) OR (Incidence[Title])) OR (Penetrance[Title]))) AND (V122i[Title/Abstract])
US	(((American[Title/Abstract]) OR (America[Title/Abstract])) OR (United States[Title/Abstract])) AND ((((Amyloidosis[Title/Abstract]) AND ((ATTR[Title/Abstract]) OR (TTR[Title/Abstract]))) AND ((((Epidemiology[Title]) OR (prevalence[Title])) OR (Incidence[Title])) OR (Penetrance[Title]))) AND (V122i[Title/Abstract]))
Clinical presentation and phenotype	
ATTR amyloidosis	
Worldwide	"Amyloidosis"[Title/Abstract] AND ("ATTR"[Title/Abstract] OR "TTR"[Title/Abstract]) AND ("Presentation"[Title] OR "Phenotype"[Title] OR "phenotypic"[Title] OR "Clinical presentation"[Title])
US	"Amyloidosis"[Title/Abstract] AND ("ATTR"[Title/Abstract] OR "TTR"[Title/Abstract]) AND ("Presentation"[Title] OR "Phenotype"[Title] OR "phenotypic"[Title] OR "Clinical presentation"[Title]) AND ("American"[Title/Abstract] OR "America"[Title/Abstract] OR "united states"[Title/Abstract])
p.Val142Ile variant-specific	
Worldwide	"V122i"[Title/Abstract] AND ("Amyloidosis"[Title/Abstract] AND ("ATTR"[Title/Abstract] OR "TTR"[Title/Abstract]) AND ("Presentation"[Title] OR "Phenotype"[Title] OR "phenotypic"[Title] OR "Clinical presentation"[Title])
US	"V122i"[Title/Abstract] AND ("Amyloidosis"[Title/Abstract] AND ("ATTR"[Title/Abstract] OR "TTR"[Title/Abstract]) AND ("Presentation"[Title] OR "Phenotype"[Title] OR "phenotypic"[Title] OR "Clinical presentation"[Title]) AND ("American"[Title/Abstract] OR "America"[Title/Abstract] OR "united states"[Title/Abstract]))
The diagnostic journey	
ATTR amyloidosis	
Worldwide	((Amyloidosis[Title/Abstract]) AND ((ATTR[Title/Abstract]) OR (TTR[Title/Abstract]))) AND (((((((Diagnosis[Title]) OR ("Diagnostic journey"[Title])) OR ("Diagnostic delay"[Title])) OR ("Delayed diagnosis"[Title])) OR ("Patient identification"[Title])) OR ("Identifying patients"[Title])) OR (Identification[Title]))
US	(((American[Title/Abstract]) OR (America[Title/Abstract])) OR (United States[Title/Abstract])) AND (((Amyloidosis[Title/Abstract]) AND ((ATTR[Title/Abstract]) OR (TTR[Title/Abstract]))) AND (((((((Diagnosis[Title]) OR ("Diagnostic journey"[Title])) OR ("Diagnostic delay"[Title])) OR ("Delayed diagnosis"[Title])) OR ("Patient identification"[Title])) OR ("Identifying patients"[Title])) OR (Identification[Title])))
p.Val142Ile variant-specific	
Worldwide	(V122i[Title/Abstract]) AND (((Amyloidosis[Title/Abstract]) AND ((ATTR[Title/Abstract]) OR (TTR[Title/Abstract]))) AND (((((((Diagnosis[Title]) OR ("Diagnostic journey"[Title])) OR ("Diagnostic delay"[Title])) OR ("Delayed diagnosis"[Title])) OR ("Patient identification"[Title])) OR ("Identifying patients"[Title])) OR (Identification[Title])))
US	((V122i[Title/Abstract]) AND (((Amyloidosis[Title/Abstract]) AND ((ATTR[Title/Abstract]) OR (TTR[Title/Abstract]))) AND (((((((Diagnosis[Title]) OR ("Diagnostic journey"[Title])) OR ("Diagnostic delay"[Title])) OR ("Delayed diagnosis"[Title])) OR ("Patient identification"[Title])) OR ("Identifying patients"[Title])) OR (Identification[Title])))) AND (((American[Title/Abstract]) OR (America[Title/Abstract])) OR (United States[Title/Abstract]))
Disease progression and severity	
ATTR amyloidosis	
Worldwide	((Progression[Title]) OR (Severity[Title])) AND ((Amyloidosis[Title/Abstract]) AND ((ATTR[Title/Abstract]) OR (TTR[Title/Abstract])))
US	(((American[Title/Abstract]) OR (America[Title/Abstract])) OR (United States[Title/Abstract])) AND (((Progression[Title]) OR (Severity[Title])) AND ((Amyloidosis[Title/Abstract]) AND ((ATTR[Title/Abstract]) OR (TTR[Title/Abstract]))))
p.Val142Ile variant-specific	
Worldwide	(V122i[Title/Abstract]) AND (((Progression[Title]) OR (Severity[Title])) AND ((Amyloidosis[Title/Abstract]) AND ((ATTR[Title/Abstract]) OR (TTR[Title/Abstract]))))
US	(((American[Title/Abstract]) OR (America[Title/Abstract])) OR (United States[Title/Abstract])) AND ((V122i[Title/Abstract]) AND (((Progression[Title]) OR (Severity[Title])) AND ((Amyloidosis[Title/Abstract]) AND ((ATTR[Title/Abstract]) OR (TTR[Title/Abstract])))))
p.Val142Ile variant mechanistic and biomarker studies	
p.Val142Ile variant-specific	
Worldwide	(((Study[Title/Abstract]) OR (Studies[Title/Abstract])) AND (((Mechanism[Title/Abstract]) OR (Mechanistic[Title/Abstract])) OR (Biomarker*[Title/Abstract]))) AND (((Amyloidosis[Title/Abstract]) AND ((ATTR[Title/Abstract]) OR (TTR[Title/Abstract]))) AND (V122i[Title/Abstract]))
Genetic testing/cascade screening	
ATTR amyloidosis	
Worldwide	((("cascade screening"[Title/Abstract]) OR ("genetic testing"[Title/Abstract])) OR (genotyping[Title/Abstract])) AND ((((Amyloidosis[Title]) OR ("Familial amyloid*"[Title])) OR (TTR[Title])) OR (ATTR[Title]))
US	((American[Title/Abstract]) OR (America[Title/Abstract]) OR (United States[Title/Abstract])) AND (((("cascade screening"[Title/Abstract]) OR ("genetic testing"[Title/Abstract])) OR (genotyping[Title/Abstract])) AND ((((Amyloidosis[Title]) OR ("Familial amyloid*"[Title])) OR (TTR[Title])) OR (ATTR[Title])))
p.Val142Ile variant-specific	
Worldwide	(V122i[Title/Abstract]) AND (((("cascade screening"[Title/Abstract]) OR ("genetic testing"[Title/Abstract])) OR (genotyping[Title/Abstract])) AND ((((Amyloidosis[Title]) OR ("Familial amyloid*"[Title])) OR (TTR[Title])) OR (ATTR[Title])))
US	((American[Title/Abstract]) OR (America[Title/Abstract]) OR (United States[Title/Abstract])) AND ((V122i[Title/Abstract]) AND (((("cascade screening"[Title/Abstract]) OR ("genetic testing"[Title/Abstract])) OR (genotyping[Title/Abstract])) AND ((((Amyloidosis[Title]) OR ("Familial amyloid*"[Title])) OR (TTR[Title])) OR (ATTR[Title]))))

*ATTR* amyloid transthyretin, *TTR* transthyretin, *US* United States

### Article selection and data extraction

Initially, article titles and abstracts were screened for eligibility (‘ATTR-CM’ and/or ‘p.Val142Ile’) and were excluded if they did not meet the above criteria. Subsequently, full texts of articles were reviewed for relevance and data were extracted into a spreadsheet to collate and summarize the study design and results. Any studies that did not meet the inclusion criteria following further review were excluded. A narrative synthesis was performed to summarize the current challenges based on the above‑mentioned themes and to determine the priority areas requiring action for each.

## Geographic, economic, and socio-economic factors in ATTR‑CM

### Current challenges

Studies have shown that over the past 40 years the reported rates of ATTR-CM and attributable deaths in the US have increased, likely reflecting (at least in part) greater disease awareness and advances in diagnostic modalities [31–33]. Overall prevalence of ATTR-CM (including the p.Val142Ile variant), heart failure, heart failure hospitalizations, and associated mortality rates were higher among certain minority populations, including individuals with African ancestry, compared with other groups [31, 33, 34]. However, the highest documented geographic prevalence of ATTR-CM, heart failure hospitalizations, and ATTR amyloidosis mortality was concentrated in the Midwest and Northeast regions, with “hotspots” around specialist amyloidosis centers [31–33]. Conversely, in the Southern states, which have a higher proportion of minority populations, prevalence of ATTR‑CM and ATTR amyloidosis mortality rates were lower than might be expected [31–33]. This pattern may reflect underdiagnosis among individuals of African ancestry, who are at the greatest risk of developing ATTR amyloidosis [31–33]. It also aligns with evidence that individuals of African ancestry with ATTR-CM are less likely to receive ATTR-targeted therapies than other patients. Notably, the same study reported that 95% of individuals of African ancestry with genetic variants carried the p.Val142Ile variant [34].

Observational evidence suggests that cost is a substantial barrier to the use of targeted therapies for ATTR-CM. In addition to differences between insurance programs in terms of ATTR-CM treatment coverage, there is also variability in prior authorization requirements and time to approval [14, 35]. Within the Medicare population, the oral TTR stabilizers tafamidis and acoramidis are covered under Medicare Part D and are associated with high out-of-pocket costs due to specialty-tier placement [14, 35, 36]. In contrast, patisiran and vutrisiran are infusion-based silencer therapies covered under Medicare Part B and, although they may require a minimum out-of-pocket cost from the patient, patisiran and vutrisiran impose substantial financial burden on the healthcare system due to their wholesale prices and the high billable costs when accounting for administration and healthcare professional service fees [14, 37–39]. The ongoing global, observational real-world DemonsTTRate study (NCT07358078) will assess healthcare resource utilization in adult patients with ATTR-CM treated with vutrisiran [40], and the ongoing, global, real-world MaesTTRo study (NCT06465810) will assess healthcare resource utilization in patients with ATTR amyloidosis of all phenotypes and on any treatment, including silencers [41].

There are certain circumstances in which Medicare coverage is more comprehensive and permits much lower copayments, including for patients qualifying for the Extra Help program. Other insurance providers may have lower copayment costs [35]. Additionally, there are charitable organizations that may provide grants to assist with the cost of ATTR amyloidosis treatments and other associated expenditure, such as travel costs [35]. However, eligibility criteria for grants and patient assistance programs are not always clear, and where support is limited to annual renewal this can create uncertainty and anxiety for patients and clinicians [14].

An analysis of data from the Centers for Disease Control and Prevention WONDER database has shown that a high Social Deprivation Index (SDI) score was associated with more advanced ATTR-CM and a higher mortality rate compared with a low SDI score [42]. In particular, social determinants such as poverty or lack of a vehicle were associated with an increased risk of presenting with later-stage ATTR‑CM, again suggesting that a lack of access to specialist centers contributes to delayed diagnosis [42].

### Priority areas of focus to combat these challenges

Several studies have suggested that specific approaches could address some of these issues. One has shown that the use of a specialist pharmacy service resulted in enrichment of treatment with tafamidis in certain groups, including those with African ancestry [43]. A specialist pharmacist, working as a member of the multidisciplinary team for the care of patients with ATTR-CM, can also provide a comprehensive review of benefits and an assessment of assistance programs [35]. An example of innovative resources for those having difficulty accessing care has been demonstrated in Jamaica [44]. The Heart Institute of the Caribbean, a central hub in Kingston, provides a full complement of inpatient and outpatient services, and oversees additional satellite clinics that provide diagnostics and outpatient care; the number of patient encounters increased from approximately 6000 per year in 2008 to approximately 22,000 per year in 2019 [44]. This model uses mobile technology for remote monitoring, with interpretation of diagnostics conducted by a cardiologist at the Heart Institute of the Caribbean hub [44].

**Fig. 1:**
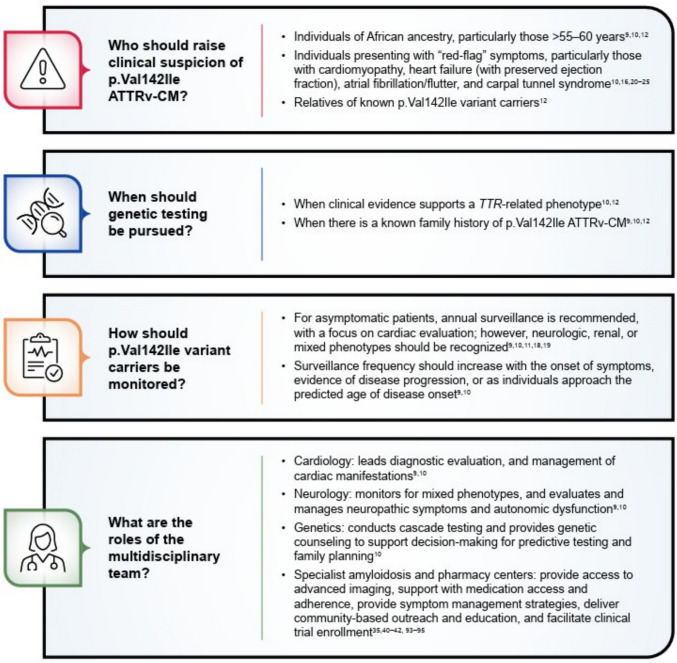
Proposed clinical workflow for p.Val142Ile ATTRv-CM. ATTRv, hereditary amyloid transthyretin; CM, cardiomyopathy

Continuing education of clinicians and raising awareness of ATTR-CM (in particular the p.Val142Ile variant) in specific communities, especially where risk is highest, is critical to breaking down some of the barriers mentioned above [45, 46]. The autosomal dominant nature of ATTRv-CM should be emphasized alongside awareness of the barriers to accessing genetic testing [14]. Leveraging existing networks such as community groups and church congregations may provide a route for outreach (Fig. 1).

## Screening approaches

### Current challenges

A diagnosis of ATTR-CM may be delayed due to the heterogeneous nature of symptoms that overlap with other diseases, e.g., hypertrophic cardiomyopathy, hypertensive heart disease, and aortic stenosis, and is an often-underrecognized cause of heart failure with preserved ejection fraction [16, 45]. In populations at risk of ATTRv-CM hypertension and heart failure tend to be common, and the prevalence of comorbid conditions such as these can undermine the diagnosis of ATTR-CM [16, 47].

When p.Val142Ile ATTRv amyloidosis is suspected, it is important to recognize that neurologic manifestations, which can precede cardiac symptoms, as well as renal or mixed neurologic and cardiac features, may occur [9, 18, 19]. Symptom assessment should therefore guide the initial evaluation and prompt a multisystem approach. Accordingly, screening should not be limited to cardiac investigations alone; clinicians should also assess for sensory neuropathy, autonomic symptoms, carpel tunnel syndrome, and changes in renal function or proteinuria.

Genetic screening offers opportunities for identifying at-risk carriers of the p.Val142Ile variant prior to overt disease development. However, current diagnostic pathways proposed by academic societies may impact identification of variants as genetic testing is listed as the last step in a step-wise approach to diagnosis of ATTR-CM [2, 48]. Following these diagnostic pathways, the absence of light chain disease and a negative nuclear scintigraphy would negate genetic testing. Earlier recognition, better screening tools, and improved diagnostic pathways are therefore needed.

In a large study (*n* > 100,000) where genetic testing was linked to electronic health records, individuals carrying the p.Val142Ile variant had significantly increased rates of atrial fibrillation and sick sinus syndrome, but only one had an existing diagnosis of ATTR amyloidosis, indicating potential underdiagnosis [49]. In another study analyzing electronic health record data from two Biobanks including individuals of African ancestry and Hispanic/Latino ethnicity, 67 individuals were identified with a clinical diagnosis of heart failure that was likely due to ATTRv-CM caused by the p.Val142Ile variant (after excluding 25 individuals with ischemic heart disease); however, only 10 (14.9%) of these individuals had been diagnosed [16].

It should be noted that a systematic genotype-first approach can be problematic due to the variable and age-dependent penetrance of the p.Val142Ile variant, and the ideal population for screening remains elusive [11, 50]. The clinical penetrance of the p.Val142Ile variant in ATTR-CM remains poorly understood, with only a subset of carriers developing overt disease, reflecting both the heterogeneity in phenotypic expression as well as the historically shorter life expectancy in carrier populations. For example, in the Screening for Cardiac Amyloidosis with Nuclear Imaging in Minority Populations (SCAN-MP; African ancestry or Caribbean Hispanic ethnicity) study of older patients with heart failure, only 39% of p.Val142Ile variant carriers (*n* = 7/18) had clinically evident ATTR-CM [11], whereas the Transthyretin Amyloidosis Outcomes Survey (THAOS) found that 96.9% of p.Val142Ile variant carriers (*n* = 32) had an associated cardiac disease (including coronary artery disease, dyspnea, heart failure, rhythm disturbance, and syncope) [51]. In a more recent US study of self-identified Black or Caribbean Hispanic individuals aged > 60 years with heart failure, approximately half (52.8%; *n* = 19/36) of p.Val142Ile variant carriers had ATTR-CM [3]. Similarly, signs and symptoms of sensory neuropathy were reported in 56.3% of p.Val142Ile variant carriers (*n* = 32) in THAOS, although distinguishing sensory neuropathy from ATTR amyloidosis versus other causes is challenging [51]; however, among US patients with the p.Val142Ile variant, the reported prevalence of neuropathy characteristics ranges from 33.8% (*n* = 25/74) in THAOS to 74% (*n* = 43/58) in a retrospective, cross‑sectional study [9, 18].

**Fig. 2:**
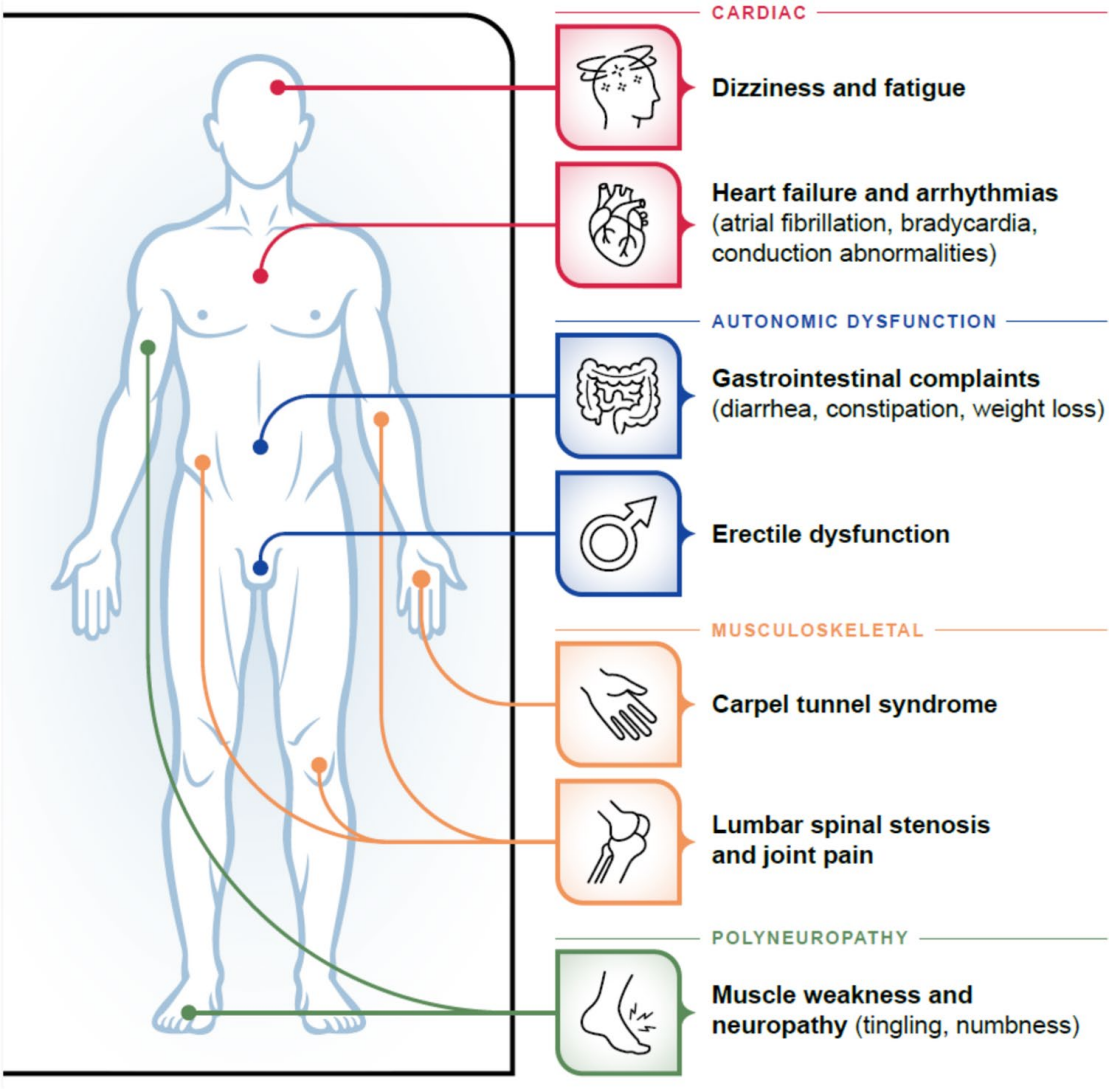
Symptoms of ATTR amyloidosis. “Red flag” symptoms of ATTR amyloidosis include: heart failure (with preserved ejection fraction), arrhythmias, dizziness, and fatigue (cardiomyopathy; red arrows); neuropathy and muscle weakness (polyneuropathy; green arrow); carpal tunnel syndrome, lumbar spinal stenosis, and joint pain (musculoskeletal; orange arrows); and gastrointestinal complaints and erectile dysfunction (autonomic dysfunction; blue arrows) [7, 14, 27, 45, 104, 105]. ATTR, amyloid transthyretin

A genetic screening program for the p.Val142Ile variant would require assembling multidisciplinary teams of experts in genetics, epidemiology, community engagement, heart failure, and amyloid care [52]. There is also the question of whether screening for *TTR* variants would be more affordable and acceptable if it were part of a panel-based approach rather than screening for variants in a single gene. Individuals with African ancestry may be understandably hesitant about a targeted screening approach and it will be essential to secure public trust through community engagement, including disease education and implications of testing on healthcare insurance [52]. In a study based in the US and Canada, patients with a family history of ATTR amyloidosis or signs consistent with ATTR-PN were offered genetic testing and 7% had a pathogenic *TTR* variant, 84% of whom had the p.Val142Ile variant [10]. Individuals with the p.Val142Ile variant predominantly self-identified as having African ancestry (82%), and many were identified in areas where no patients have previously been reported [10]. This suggests that, with the right multidisciplinary team and strategies in place to overcome any hesitancies, targeted genetic screening of at-risk patients based on family history and “red flag” symptoms (Fig. 2) has the potential to be a feasible approach [52, 53].

### Priority areas of focus to combat these challenges

Although evidence is limited, the use of a standardized genetic test requisition form could facilitate earlier identification of individuals with ATTRv-CM and provide information on geographic and demographic disparities in testing, particularly among underrepresented minority populations [54]. Furthermore, performing genetic testing earlier in the diagnostic pathway, particularly in at-risk populations, has been proposed to improve identification of patients, and subsequently improve clinical outcomes, in response to timely initiation of treatment plans [54]. Collaboration with genetic counselors is also advised before and after testing to ensure the patient has a full understanding of the implications for themselves and at-risk family members [14]. However, challenges to cascade testing remain, including access, living with the knowledge of being a p.Val142Ile variant carrier, and the potential impact on asymptomatic carriers’ ability to obtain medical or life insurance [33, 35].

A number of biomarkers have been suggested to simplify screening for the p.Val142Ile variant. Significantly lower TTR (serum prealbumin) levels have been found in patients of African ancestry or Caribbean Hispanic ethnicity with heart failure and the p.Val142Ile variant compared with controls or those with ATTRwt [11]. Retinol-binding protein 4 has also been suggested as a sensitive marker of ATTR-CM associated with the p.Val142Ile variant in individuals with African ancestry when combined with selected echocardiographic and electrocardiogram parameters [55]. However, it is important to note that serum TTR and retinol-binding protein 4 are nonspecific diagnostic markers as their respective concentrations can be affected by conditions unrelated to ATTR amyloidosis [56–59]. As the circulating levels of retinol-binding protein 4 and TTR biomarkers are highly correlated [60], the benefit of testing both remains uncertain. Additionally, the reported association of B-type natriuretic peptide and presence of the p.Val142Ile variant is inconsistent across studies [9, 55]. It has also been suggested that cardiac structure and function may differ between p.Val142Ile variant carriers and noncarriers [61], and between p.Val142Ile ATTRv-CM and ATTR-CM caused by other *TTR* variants [62]. Markers of left atrial myopathy may uniquely associate with disease severity in carriers [23]. In a study investigating the natural history and cardiovascular burden of the p.Val142Ile variant among US carriers with African ancestry, it was reported that carriers consistently lived 2–2.5 years fewer than noncarriers aged between 50 and 85 years, suggesting that increased biomarker screening from the age of 50 years could be beneficial given the clear age-dependent penetrance of the variant [12, 63].

The advent of artificial intelligence (AI) may prove beneficial in the diagnosis of ATTR-CM. A machine learning algorithm based on semiautomated cardiac magnetic resonance imaging data and clinical variables showed high accuracy in differentiating between patients with ATTR-CM and healthy controls [64]. A study conducted across the Yale–New Haven Health System used a library of echocardiographic studies to train deep learning algorithms for use in lower-quality point-of-care cardiac ultrasonography; in the emergency care setting, enhanced identification of hypertrophic CM and ATTR-CM was observed [65]. These findings suggest the utility of AI to permit the use of modalities that are available in low‑resource settings to provide an accurate diagnosis of ATTR-CM [65]. Given the racial disparities in p.Val142Ile ATTRv-CM, it is crucial that the development and training of AI algorithms include data from minority populations and patients with p.Val142Ile ATTRv-CM to mitigate systematic bias in AI applications [66].

Interpersonal connections (such as those between patients and healthcare professionals), a lack of trust in the healthcare system, and poor health literacy also present barriers to the testing of asymptomatic individuals, as well as their confidence in asking questions. Access to information, especially information that is available online, may also be an issue in certain populations.

Importantly, while genotype-first screening may help identify at-risk p.Val142Ile variant carriers and facilitate earlier diagnosis, its implementation is complicated by several uncertainties mentioned previously. Current evidence, although limited, supports a targeted approach in high-risk groups, accompanied by multidisciplinary support and careful patient education, rather than a broad, population-wide genotype first approach (Fig. 1). These uncertainties should be central to any discussion of genetic screening strategies for ATTR-CM, ensuring that recommendations remain aligned with the evolving evidence base. 

## Monitoring symptom progression

### Current challenges

Even when carriers of ATTRv-CM are identified, the optimal clinical surveillance remains uncertain in terms of frequency, imaging, and blood testing. Adding complexity, many centers may not have the capability to offer comprehensive workup and imaging in all carriers [50]. Given the age-dependent penetrance of the p.Val142Ile variant, carriers would need to be seen regularly by an experienced care team [52].

Individuals with p.Val142Ile ATTRv-CM (or p.Val142Ile variant carriers) may have a higher prevalence of heart failure and other clinical confounders that cause cardiovascular mortality and morbidity [16, 19–21]. The UK Biobank database of 500,000 individuals aged 40–69 years found a higher-than-expected frequency of *TTR* variants within a population composed predominantly of individuals of European ancestry with no known clusters of high prevalence for this condition. Among these, the p.Val142Ile variant was the most frequently observed [67]. Individuals with p.Val142Ile variant carrier status were associated with a higher risk of incident heart failure and cardiac conduction disease when compared with noncarriers [67]. Compared with ATTRwt-CM, individuals with p.Val142Ile ATTRv-CM experience significantly faster disease progression, due to deterioration in heart structure and function, and poorer survival [23, 68, 69]. Evidence suggests that the p.Val142Ile variant is also associated with more advanced disease, a more rapid decline in renal function, and poorer survival in patients with ATTRv-CM compared with those carrying other *TTR* variants [19, 70–72].

Neurologic symptoms including neuropathic pain and numbness are often reported more frequently with p.Val142Ile ATTRv-CM compared with patients with ATTRwt-CM, even though individuals with p.Val142Ile ATTRv-CM and ATTRwt-CM have similar cardiac profiles [9]. However, ATTRv-PN may be overlooked for a variety of reasons: misdiagnosis with diabetic peripheral neuropathy, clinical inertia, and assessing penetrance of the p.Val142Ile variant on cardiac imaging alone [11, 18]. It should also be noted that the phenotype of ATTRv-associated disease (all variants) can evolve over time, although the extent and clinical significance of this for individuals carrying the p.Val142Ile variant have yet to be fully characterized [73]. Follow‑up in these patients should therefore extend beyond cardiac parameters alone, similar to at initial screening, with clinicians routinely evaluating for evolving sensory neuropathy, autonomic symptoms, carpel tunnel syndrome, and changes in renal function or proteinuria. A multisystem monitoring strategy enables earlier detection of disease progression, supports timely adjustment of therapy, and ensures that non-cardiac manifestations, which may affect quality of life, are not overlooked.

### Priority areas of focus to combat these challenges

Consensus groups have proposed guidelines for the diagnosis and management of ATTR amyloidosis; however, criteria for establishing disease progression and when a switch in therapy may be needed are poorly defined [2, 13, 14, 74, 75]. Additionally, the guidelines lack recommendations for monitoring patients with ATTR mixed [2, 74]. It has been recommended that ATTRv carriers should undergo routine evaluation beginning 10 years earlier than when the youngest member of the family developed ATTRv-CM or ATTRv-PN [13, 76]. Proposed monitoring for these individuals is on a yearly basis, possibly starting around age 50, but with more frequent follow-up as they approach the predicted onset of symptomatic disease [13]. Homozygous individuals have much earlier disease presentation, however, and likely need to be assessed at earlier ages [12, 77]. Tests may be directed at the expected phenotype associated with the specific *TTR* variant and involve a multidisciplinary team (Fig. 1) [13]. Due to the presence of early cardiac conduction abnormalities in the UK Biobank cohort, it has been suggested that echocardiography, cardiac magnetic resonance imaging, and electrocardiography be used to identify early transition to ATTR-CM [50]. In the SCAN-MP screening study of patients with heart failure, those with a clinical diagnosis of p.Val142Ile ATTRv-CM demonstrated significantly higher N‑terminal pro-B-type natriuretic peptide, but lower prealbumin, shorter 6-min walk distance, and lower stroke volume and cardiac output, than carriers of the p.Val142Ile variant without a diagnosis of ATTR-CM, suggesting that these parameters could be used to increase clinical suspicion in known p.Val142Ile variant carriers [11]. Minimal consensus criteria to establish the onset of symptomatic ATTR amyloidosis have been published [13].

## Tailoring treatment strategies

When p.Val142Ile ATTRv amyloidosis is confirmed or strongly suspected, treatment decisions should be tailored to the patient’s clinical history and trajectory of symptom progression. Although cardiomyopathy is the predominant feature in p.Val142Ile ATTRv‑CM, neurologic, renal, or mixed manifestations may influence both the choice and timing of therapy [6, 72, 73]. Management should therefore integrate cardiac‑directed treatments with supportive measures for neuropathic or autonomic symptoms, and careful consideration of medications in the context of renal function (Fig. 1). This personalized, multisystem approach is particularly important given the limited treatment options, evidence, and recommendations available, especially for patients with ATTR mixed phenotypes [74], and provides the foundation on which current and emerging therapeutic options must be evaluated.

### Approved treatments

Phase 3 randomized controlled trials have led to the approval of numerous disease‑modifying therapies. Stabilizers such as tafamidis and acoramidis bind to the TTR protein, preventing dissociation of tetramers into amyloidogenic monomers [78, 79]. Both agents are approved for the treatment of ATTR-CM in the US, specifically [80, 81]. Vutrisiran (approved for ATTR-CM and ATTRv-PN in the US) [82], along with patisiran and eplontersen (both approved for ATTRv-PN in the US) [83, 84], are *TTR* gene silencers that target messenger RNA, reducing TTR synthesis and subsequently the availability of amyloidogenic monomers [85–87].

### Treatments in ongoing trials

The ongoing phase 3 CARDIO-TTRansform study (NCT04136171) will evaluate the efficacy of eplontersen compared with placebo in patients with ATTR-CM who are receiving available standard of care [88]. A new *TTR* silencer, nucresiran, is also in phase 3 trials for patients with ATTR-CM (NCT07052903) [89] and ATTRv-PN (NCT07223203) [90]. Together, these trials offer an opportunity to enhance our understanding of the broad and diverse patient population, as well as key patient subsets, which will in turn support future diagnosis and the provision of appropriate treatments to manage symptom progression.

The potential benefits of dual therapy with a protein stabilizer and gene silencer are as yet unclear [91]. In the HELIOS-B study, a subgroup of patients with ATTR-CM (49 patients with the p.Val142Ile variant) receiving vutrisiran were also treated with tafamidis at baseline. There was an improvement in the primary endpoint (composite all-cause mortality and recurrent cardiovascular events) and all-cause mortality alone in this population versus placebo [87, 92]. However, in an analysis of real-world cohort data, combination therapy with a gene silencer and a protein stabilizer conferred no additional benefit over monotherapy in terms of cardiovascular hospitalizations and all-cause mortality [91].

### Prophylactic concepts

The ongoing phase 3 ACT-EARLY (Acoramidis Transthyretin Amyloidosis Prevention Trial in the Young) study (NCT06563895) will test the hypothesis that prophylactic acoramidis treatment can prevent or delay the onset of ATTR-CM or ATTR-PN disease in asymptomatic patients harboring a pathogenic ATTRv variant [93, 94]. Findings will be of particular interest for asymptomatic p.Val142Ile variant carriers.

### Emerging gene and antibody-based therapies

Gene therapies are at an early stage of clinical development, although one CRISPR/Cas9 agent (nexiguran ziclumeran, previously known as NTLA-2001) has shown promising reductions in serum TTR in patients with ATTRv-CM (including the p.Val142Ile variant) [95] and ATTRv-PN (patients with the p.Val142Ile variant were not assessed) [96]. Two phase 3 studies (MAGNITUDE and MAGNITUDE-2), investigating the effects of nexiguran ziclumeran in patients with ATTR-CM (NCT06128629) [97] and ATTRv-PN (NCT06672237) [98], are ongoing. Antibody depleters are also promising therapeutic agents for ATTR amyloidosis. The ongoing phase 3 CLEOPATTRA trial will investigate the effects of coramitug (NNC6019-0001) on cardiovascular outcomes in patients with ATTR-CM (NCT07207811) [99].

Although effective therapies for ATTR-CM and ATTR-PN are now available access to such treatments needs to be ensured; for example, the cost and logistics of traveling to centers for infusions need to be considered [42]. Furthermore, healthcare teams will need to contend with complex processes in prescribing therapies [52]. These issues will become even more significant if care shifts toward a preventive treatment approach.

## Clinical trial enrollment

Despite the high burden of p.Val142Ile ATTRv-CM in individuals with African ancestry, most clinical trial data are from North America and Europe, with a minority of participants with African ancestry or those with the p.Val142Ile variant [100]. ATTR-CM trials often do not include African countries as study centers, nor do they have explicit goals for recruitment of patients with African ancestry or who carry the p.Val142Ile variant [100]. In the SCAN-MP study (where the average proportion of individuals with African ancestry or Caribbean Hispanic ethnicity was approximately 40%), the most successful recruitment strategy was via direct contact from the patients’ primary cardiovascular teams [101]. Future trials should model recruitment and enrollment on past trials that have evaluated outcomes in patients with ATTRv-CM and the p.Val142Ile variant specifically, and trials that have enrolled a high number of minority populations. Strategies could include direct initial contact from the patients’ primary care teams and educational outreach, targeting sites in communities with high minority representation, leveraging existing registries, addressing language barriers that might prevent minority patients from enrolling, and providing appropriate, culturally sensitive multimedia information to improve trust [16, 101–103]. Target recruitment numbers for participants from minority populations are needed for findings to be generalizable to the real-world population.

## Conclusions

Despite increasing recognition of the underdiagnosis and misdiagnosis of ATTR amyloidosis, particularly among minority populations, including individuals with African ancestry who experience worse outcomes and a poor prognosis, a number of challenges remain: (1) a lack of access to specialist treatment centers and the financial burden of accessing available healthcare; (2) symptom heterogeneity that overlaps with other diseases and the variable penetrance of variants such as p.Val142Ile, which have the potential to delay diagnosis and initiation of appropriate treatment; and (3), a poor understanding of ATTRv amyloidosis progression and inadequate treatment guidelines. To counter these challenges facing clinicians and individuals at risk of ATTRv amyloidosis, the priority areas for focus are: (1) targeted education of clinicians and local communities in “hotspot” regions to ensure equitable access to, and uptake of, genetic screening (including the p.Val142Ile variant) and inclusive clinical trial enrollment; (2) defining appropriate clinical surveillance strategies for carriers of ATTRv amyloidosis and specifically the p.Val142Ile variant, (3) innovative resourcing of multidisciplinary teams to provide care and remote monitoring by clinicians to enrich patient treatment opportunities, and (4) further research to validate the clinical utility of potential biomarkers and to identify additional candidates. In turn these focus areas will underpin refinement of evidence-based treatment guidelines and strategies for optimal patient management in ATTRv amyloidosis.

## Data Availability

No datasets were generated or analysed during the current study.
